# Light self-focusing in the atmosphere: thin window model

**DOI:** 10.1038/srep30697

**Published:** 2016-08-02

**Authors:** Irina A. Vaseva, Mikhail P. Fedoruk, Alexander M. Rubenchik, Sergei K. Turitsyn

**Affiliations:** 1 Institute of Computational Technologies, Siberian Branch of the Russian Academy of Sciences, 6 Ac. Lavrentjev Avenue, Novosibirsk, 630090, Russia; 2 Novosibirsk State University, 2 Pirogova Street, Novosibirsk, 630090, Russia; 3 Lawrence Livermore National Laboratory, Livermore, California 94550, USA; 4 Aston Institute of Photonic Technologies, School of Engineering and Applied Science, Aston University, Birmingham, B4 7ET, UK

## Abstract

Ultra-high power (exceeding the self-focusing threshold by more than three orders of magnitude) light beams from ground-based laser systems may find applications in space-debris cleaning. The propagation of such powerful laser beams through the atmosphere reveals many novel interesting features compared to traditional light self-focusing. It is demonstrated here that for the relevant laser parameters, when the thickness of the atmosphere is much shorter than the focusing length (that is, of the orbit scale), the beam transit through the atmosphere in lowest order produces phase distortion only. This means that by using adaptive optics it may be possible to eliminate the impact of self-focusing in the atmosphere on the laser beam. The area of applicability of the proposed “thin window” model is broader than the specific physical problem considered here. For instance, it might find applications in femtosecond laser material processing.

A ground based pulsed laser system is a promising way to mitigate the growing space debris problem^1^. Following decades of space exploration an ever-growing cloud of more than twenty-two thousands pieces of space junk are now orbiting the Earth, posing a serious treat to satellites and corresponding technologies and services. The pulsed laser ground based system for the debris removal was proposed and evaluated in nineties^2^. Today, the progress in laser and optical engineering makes the system design within the reach of modern technology^1^,^3^. Such debris-removal laser system with an ultra-long reach (on the scale of the distance to the orbit) must exploit ultra-high power beams with a power exceeding the self-focusing threshold by more than three orders of magnitude. The propagation of a pre-focused (spatial pre-chirping) high-power laser beams through the atmosphere is very different from studies of conventional self-focusing and filamentation in air or gases. The important new feature is that such a pre-focused beam may be free of filamentation even for very high input power^1^,^4^. Recently it was demonstrated that for the typical parameters of a laser pulse, self-focusing in the atmosphere can impair the laser beam quality, decreasing the laser intensity on the debris and degrading the system performance^4^. To some extent, the effect of the self-focusing can be compensated by pre-defocusing of the initial beam. However, the optimization of the pre-focusing requires complex and time-consuming modeling.

Here we demonstrate that the situation can be accurately modeled within the “thin window” (TW) approximation^5^. When the thickness of the atmosphere is much smaller than the focusing length, propagation through the atmosphere results in phase distortion only. Due to exponential decay of the nonlinear effects, the remaining propagation (beyond the atmosphere) is effectively linear, greatly simplifying modeling. Effectively this can be treated as split-step approach with just one nonlinear and one linear steps to model beam propagation. We show here that the TW model prediction is in excellent agreement with solutions of the exact nonlinear Schrödinger equation (NLSE). Using the TW model we are able to calculate the optimal focusing conditions to compensate maximally the aberration produced by the self-focusing in the atmosphere. We have also calculated the reduction in the peak intensity at the focal plane due to the non-compensated aberrations and the displacement of the focal point.

The high accuracy of the TW model has an important practical application. Using adaptive optics one can apply an initial phase pre-distortion, which compensates the nonlinear phase changes. We will demonstrate that as a result one can have an almost perfect Gaussian beam at the atmospheric exit, and the detrimental effects of self-focusing can be eliminated to a great extent.

For most of the applications considered here, it is sufficient to know only the intensity in the spot center and the spot size^1^ (effectively, even knowledge of the ratio of these values for an aberrated beam compared to the ideal Gaussian one). We present simple analytical expressions for these important practical parameters based on TW model and verify their applicability through numerical modeling using NLSE.

The operations of the complex laser system considered here depend on numerous parameters, such as pulse energy, focusing mirror size, focal distance, laser system altitude, and others. As a result, the direct system optimization, even though possible, requires much time-consuming effort. Our model indicates that there exists a scaling in the problem, determined by a combination of the key parameters. This scaling makes it possible to relate the results for different sets of parameters and greatly reduces the optimization efforts.

## Thin Window (TW) model

The main effects accompany the powerful beam propagation in atmosphere were discussed in mid seventies (see e.g. book^6^). The processes affected ground based space debris cleaning, including the non-linear ones, were evaluated during Orion project^2^. The most important effects includes the self-focusing, Raman scattering and the scattering by the atmosphere turbulence. For operating system the all detrimental effects must be small and can be evaluated independently. In present paper we will discuss the self-focusing effects only. The effects of turbulence and Raman scattering can be important (see discussion in^4^) and limits the system operational parameters.

The analysis of the ground-based laser system for space debris cleaning indicates that the laser power must greatly exceed, by a factor of 1000–5000, the critical power *P*_*cr*_ for self-focusing in the atmosphere^3^,^4^. The optimal parameters for the laser system for debris cleaning were discussed in^3^. Let us consider a specific example used in^3^ in which wavelength *λ* = 1 μm debris orbit height *L* = 1000 km mirror diameter *D* = 3 m and beam quality parameter *M*^2^ = 2 This value of *M*^2^ can be achieved for high-energy lasers by using spatial filters and adaptive-optic systems. For this case the spot size on the target *r* = 34 cm the required pulse energy is 
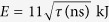
 and pulse power 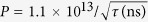. For the optimal point of view from laser technology^3^, pulse duration ~3 ns, the required power *P* = 6TW much higher than the critical power for self-focusing in atmosphere *P*_*cr*_ = 4GW.

**Figure 1:**
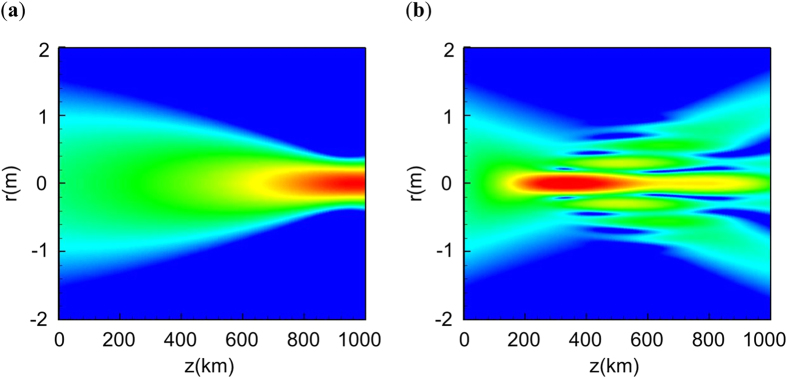
The normalized intensity distribution I(r, z)/I(0, 0) 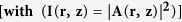. (**a**) *P*_*in*_/*P*_*cr*_ = 100, *C* = 5.93. (**b**) *P*_*in*_/*P*_*cr*_ = 5000, *C* = 5.93. These conditions correspond to a focusing distance *L* = 1000 km for the linear propagation.

The self-focusing length in this situation is much longer than the atmospheric thickness, and nonlinear effects produce only phase aberrations, which during the following long (about 1000 km) free propagation to the debris can greatly modify the beam (see Fig. 1). One can see that as a result of the nonlinear focusing in the atmosphere, the intensity peak moves back to the ground, and at high power when filamentation becomes important, the transverse beam shape is far from Gaussian. Let us consider the problem in more detail.

The propagation of the laser beam is described by the nonlinear Schrodinger equation (see e.g.refs 4,5), i.e.


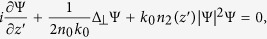


where Δ_⊥_ is the two-dimensional Laplacian operator. The analysis in^3^ demonstrates that the optimal pulse length, based on physics and engineering considerations, is on the order of a few nanoseconds. For this order of pulse length, temporal dispersion can be neglected in the main order of the considered effects. The inhomogeneity of the density must be taken into account in the nonlinearity only^1^,^3^,^4^.

Here we consider a laser beam propagating vertically (relative to the ground). This is not very different from the optimal angle for the interaction with debris, which is ~30 degrees from the vertical^3^. The assumption of perpendicular propagation is not critically important, but it simplifies the presentation. It is customary to introduce dimensionless variables:


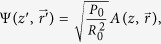
 where dimensionless *z* = *z*′/*L*_*D*_ and 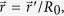

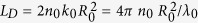
, *k*_0_ = 2*π*/*λ*_0_, *λ*_0_ = 1.06 μm, *k*_0_ = 5.93 μm^−1^, *n*_0_ = 1.0, *n*_2_(0) = 4.2 × 10^−19^ cm^2^/W. Here *z* = 0 corresponds to sea level. We assume the commonly used exponential density dependence with the atmosphere height *Z*_0_ = 6km, *n*(*z*′)/ *n*(0) = exp(−*z*′/*Z*_0_). The nonlinear effects decay with height as *n*_2_(*z*′) = *n*_2_(0)·exp(−*z*′/*Z*_0_)^1^,^4^. Here a normalization parameter *R*_0_ corresponds to the initial radius of the beam. The power is normalized by the value 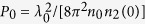. Debris is located at the distance *L*. The resulting normalized equation has the form:


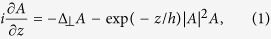


where *h* = *Z*_0_/*L*_*D*_. For *R*_0_ = 1 m and the parameters given above, we have *L*_*D*_ = 11855 km, *P*_0_ = 0.339 GW and *P*_*cr*_ = 4*πP*_0_ = 4.258 GW for a Gaussian input beam.

It is possible to show that the fastest growing perturbations resulting in filamentation are axisymmetric^7^, and that filamentation, at least initially, breaks the beam into ring-like structures. The formation and break-up of the ring structure is the well-documented pathway to Gaussian beam filamentation^8^, and beam propagation can be described in the main order within the axisymmetric version of Eq. (1). Transition from the ring filamentation to the isolated filaments were demonstrated in^9^.

The problem is characterized by three dimensionless parameters: the ratio *p*_*in*_/*P*_*cr*_ of the input beam power *P*_*in*_ to the critical power, the initial beam pre-focusing parameter *C* (see below), and a parameter *h* characterizing decay of the nonlinear effects with distance, typically 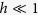. Let us consider the propagation of an initially Gaussian laser beam. On the surface at *z* = 0:


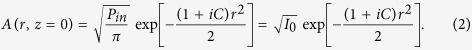


Here *P*_*in*_ is the normalized input power of the laser beam, the dimensionless parameter 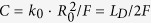 is the initial beam pre-focusing parameter. 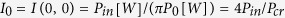. *F* has the meaning of a focal distance that in this case is a debris height *L*. Note that this is a true statement only when 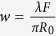
is much smaller than *R*_0_ that is not always a case for long space propagation, as we will see below, e.g. in Fig. 1b. Therefore, dimensional initial pre-focusing is given by *C* = *L*_*D*_/2*L*.

We would like to stress that the problem under consideration, though similar in terms of the basic equations to numerous self-focusing studies (see e.g. refs 5,10 and references therein), is rather different in terms of underlying physics. The nonlinearity exponentially decays with propagation. Therefore, system is strongly inhomogeneous with spatially decreasing nonlinear effects. Our laser beam has a much larger spot size (over 1 m). The self-focusing length 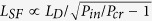 is much longer than the thickness of the atmosphere. This displaces the self-focusing (beam collapse) point far beyond the atmosphere. In other words, we consider here light propagation over a finite distance (the thickness of the nonlinear layer), with the focusing point located beyond this region, where the propagation is linear. In this case the self-focusing effect compresses the beam, but without the catastrophic collapse of all the energy into a small volume. Numerical modeling^4^ strongly indicates that for the problem examined here, even for input powers well above the critical power for self-focusing, the beam can maintain its integrity and is focused as a whole.

Let us apply now in this context the thin window model, following 5,10,11,12. We replace the numerical calculation of the propagation described by Eq. (1) by just one an effective nonlinear step and following linear propagation. After propagation of the short distance z_1_ the impact of the nonlinear term in Eq. (1) can be formally written as:


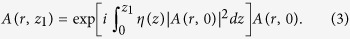


When the beam propagates up to atmospheric boundaries, the impact of the diffraction can be estimated as ~*z*_1_ ~ *h* ~ *Z*_0_/*L*_*D*_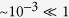, and the effect of the nonlinearity can be estimated as *hP*_*in*_/*P*_*cr*_, which can be larger than unity. As the nonlinearity is decaying exponentially, the initial propagation stage is the most important and after it, propagation is linear with a modified phase. Our approach is based on the observation that during propagation through the atmosphere, with high accuracy we can disregard the mutual impact of the nonlinearity and diffraction. Taking into account only the phase distortion due to nonlinearity, the laser field after propagation through the atmosphere is given by Eq. (3).

For a Gaussian initial beam we have the explicit description of the field:


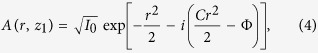


where 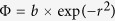, 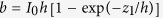, 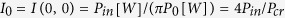. The parameter *b* has the meaning of the nonlinear phase shift scale and is the analog of *B* integral used by laser designers for the evaluation of nonlinear effects in the uniform windows^13^. As we will show below, it is important for understanding the ensuing linear dynamics.

The value of *z*_1_ that is certainly a critical parameter of the model is approximately a several times h; the optimal choice of *z*_1_ will be further discussed below, after comparison with numerical modeling results. It is clear that there must exist an optimal value of *z*_1_, since for small *z*_1_ we cut out the part of the atmospheric propagation, and when *z*_1_ is too large the free propagation will modify the solution (the window is no longer thin). Due to the exponential dependence on *z*_1_ in (4), the optimal value is about a few atmospheric thicknesses. Let us make some estimate. We require the maximal phase deviation at *z* = *z*_1_ to be different from that at the infinite *z*_1_ by less than α radians. In this case, *z*_1_ is given by *z*_1_/*h* = In(*I*_0_*h*/*α*). Due to the logarithmic dependence, *z*_1_ depends only weakly on the laser power and the choice of α. For *α* = 0.01, *z*_1_ in dimensional units increases from 36 km to 45 km when the power changes from *P* = 1000 *P*_*cr*_ to 5000 *P*_*cr*_, and the choice of *z*_1_ does not strongly affect the results. The following results will be presented for some particular *z*_1_ values, and the impact of the choice of *z*_1_ on the results obtained by the TW model will be discussed in detail in Appendix below.

**Figure 2:**
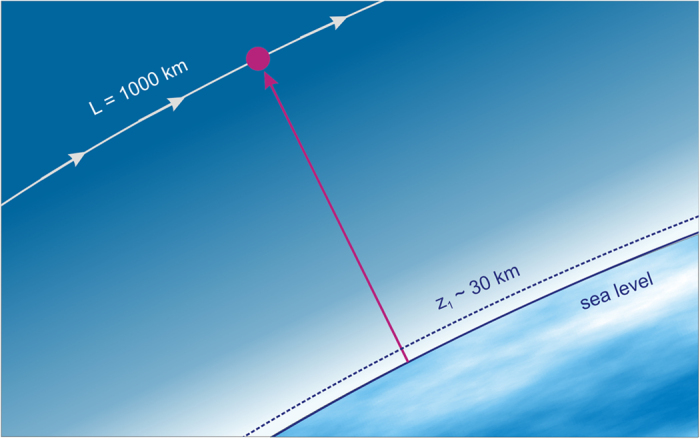
Illustration of the thin window model.

After the beam exits the atmosphere, it undergoes almost free linear propagation (see Fig. 2). From the Eq. (4) we see that the phase is not quadratic, and the ensuing beam propagation is not described by the simple formula available for the focused Gaussian input. The curvature of the phase corresponds to an additional focusing, and the atmosphere serves as a focusing astigmatic lens. As a result, the maximal field intensity is reached before the linear focal plane. By varying the pre-focusing parameter *C* (or even adjusting the initial beam phase to compensate more accurately for the nonlinear phase aberration using adaptive optics) we can partially compensate for the propagation through the atmosphere^4^.

To evaluate the field in the focal plane consider the solution of the linear problem [Eq. (1) without nonlinear term):


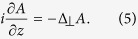


The solution of Eq. (5) at a distance *z* can be expressed using a well-known Green’s function in terms of the field at *z* = *z*_0_, *A*(*x*,*y*,*z* = *z*_0_)=*A*_1_(*x*,*y*), namely:


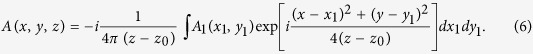


Assuming beam at *z* = *z*_0_ to satisfy Eq. (4) we can write down explicitly the focusing part:


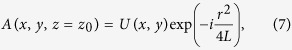


where *r*^2^ = *x*^2^ + *y*^2^ and 
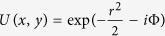
.

Substitution of this expression into Eq. (6) yields


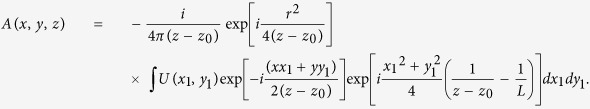


We see that at the focal plane *z* = *z*_0_ + *L*, the Fresnel quadratic terms are cancelled and the electric field is proportional to the Fourier transform of the field on the boundary^3^


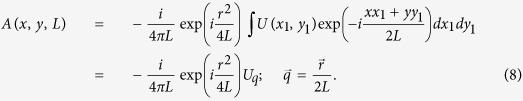


We focus in what follows only on the axisymmetric beam propagation. The general solution of the linear problem (6) for the axisymmetric case can be transformed to the form:


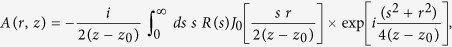


where 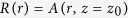. Applying the explicit extraction of the focusing factor
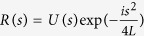
, we see that the field at *z *= *z*_0_ + *L* is given by the Fourier transform of the field at *z* = *z*_0_. To keep a focal point close to the same distance *z* = *L*, we can either account for the effective linear phase change during the propagation from *z*_0_ = 0 to *z* = *z*_1_ or consider linear propagation from *z* = 0 with a phase modified due to nonlinear effect – an effective split step method with just one nonlinear and one linear steps. Here, for simplicity, we use the later (use *z*_0_ = 0) and demonstrate an excellent agreement between this simplified TW approach and the full numerical modelling of NLS equation.

As a matter of fact, in the practical applications, one usually needs only a subset of this full field description, namely, an information about the intensity on the target: the intensity in the spot center and the average spot size. Therefore, the description can be further simplified.

Assuming that the field at *z* = *z*_0_ is 
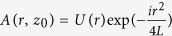
, the electric field on the target in the focal plane can be presented as a Fourier transform of that in Eq. (8)^14^. Namely, the field in the spot center is given by the expression:


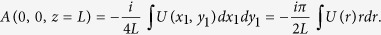


It is customary to use the Strehl ratio S, the ratio of the intensity in the center to that produced by the linear evolution of the Gaussian beam (2):


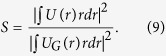


Recalling that 
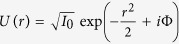
, 
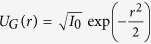
, we can calculate the Strehl ratio explicitly:


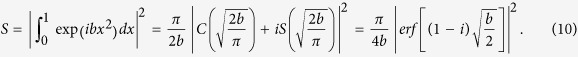


The integral estimate of the square of the spot size on the target can be calculated in a similar way as follows:


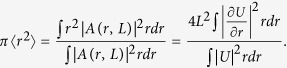


Similarly, it is convenient to calculate analytically the beam quality parameter *M*^2^, the ratio spot size square (9) to the value calculated for the Gaussian beam


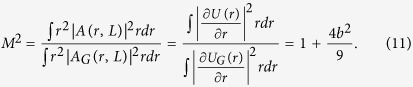


## Results and Discussion

### Comparison with the direct numerical modelling

**Figure 3:**
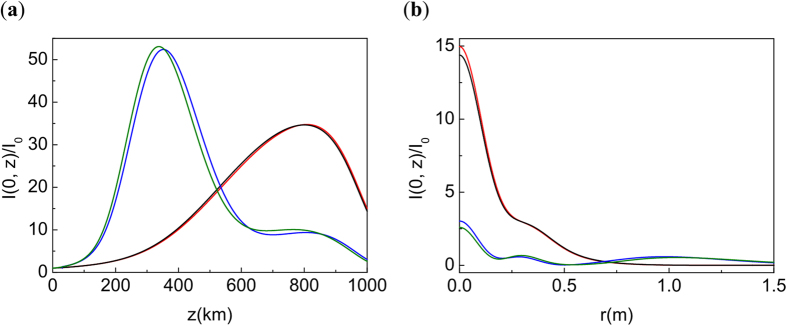
The comparison of the solution of NLSE (1) with the TW model (4) for *z*_1_ = 30 km. (**a**) The peak intensity distribution along *z.* (**b**) The radial intensity profile at the initial focal point *L* = 1000 k. Black line: NLSE, *P*_*in*_/*P*_*cr*_ = 1500. Red line: TW, *P*_*in*_/*P*_*cr*_ = 1500. Green line: NLSE, *P*_*in*_/*P*_*cr*_ = 5000. Blue line: TW, *P*_*in*_/*P*_*cr*_ = 5000.

**Figure 4:**
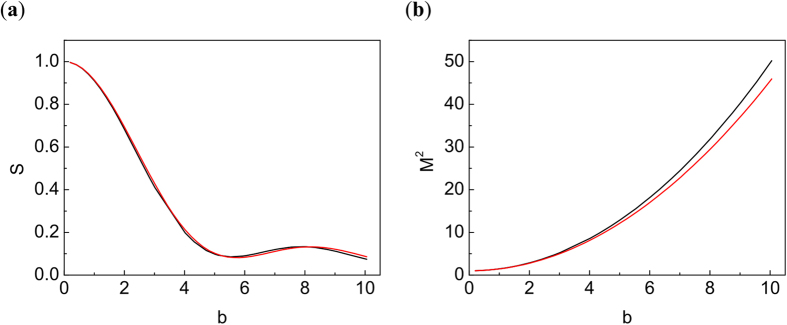
(**a**) Comparison of Strehl ratio computed from NLSE (1) and TW model (10). (**b**) Comparison of the beam quality parameter *M*^2^ from formula (11) with results of direct modeling using the NLSE. Black line: NLSE. Red line: TW for *z*_1_ = 30 km.

**Figure 5:**
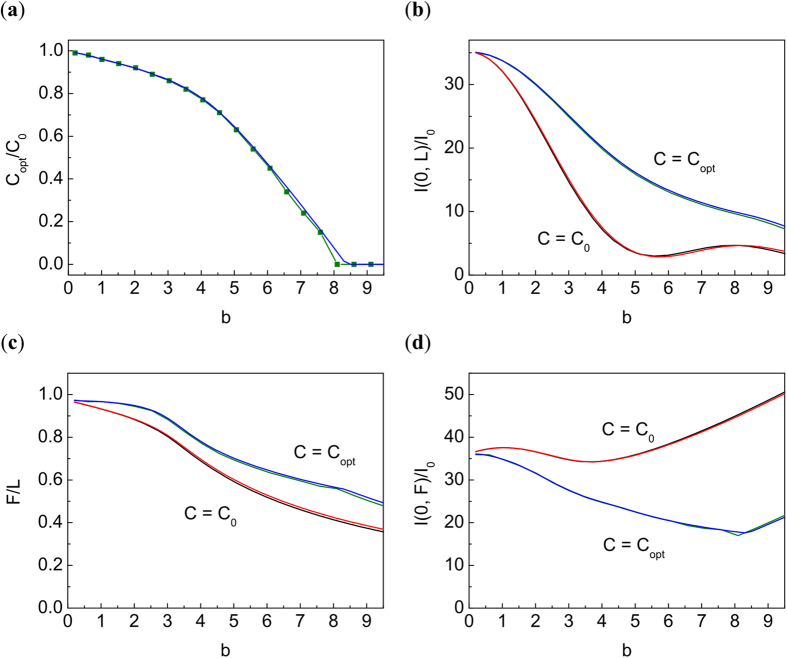
(**a**) The optimal chirp *C*_opt_ normalized by the initial one *C*_0_ as a function of *b*. Here 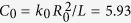. (**b**) The normalized intensity at the initial focal point *L* = 1000 km depending on *b* for the initial and optimal chirps. (**c**) New focusing length *F*/*L* depending on *b* for the initial and optimal chirps. (**d**) The normalized intensity at new focal points 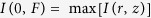 depending on *b* for the initial and optimal chirps. The maximum value of *b* corresponds to *P*_*in*_/*P*_*cr*_ = 5000. Black line: NLSE, *C* = *C*_0._ Red line: TW, *C* = *C*_0_. Green line: NLSE, *C* = *C*_*opt*_. Blue line: TW, *C* = *C*_*opt*_.

**Figure 6:**
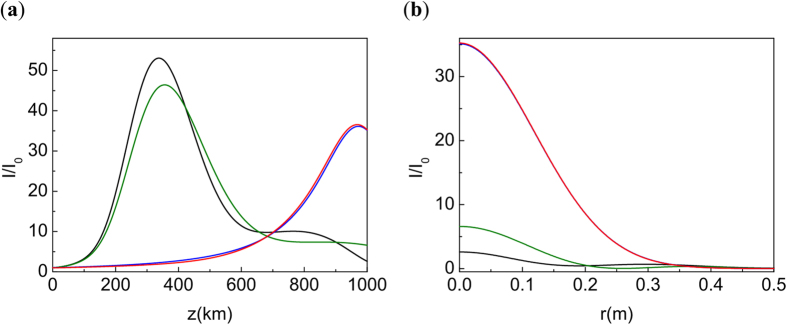
(**a**) Intensity distribution along *z*. (**b**) Radial distribution at the initial focal point *L* = 1000 km. *P*_*in*_/*P*_*cr*_ = 5000. Black line: NLSE with *C* = 5.93. Green line: NLSE with *C* = *C*_*opt*_. Red line: NLSE with *C* = 5.93 and pre-imposed phase (12). Here *z*_1_ = 30 km. Blue line: Propagation of Gaussian beam in linear case. Note that the effective focal point is shifted from *z* = *L*, see the explanation and discussion in^16^.

**Figure 7:**
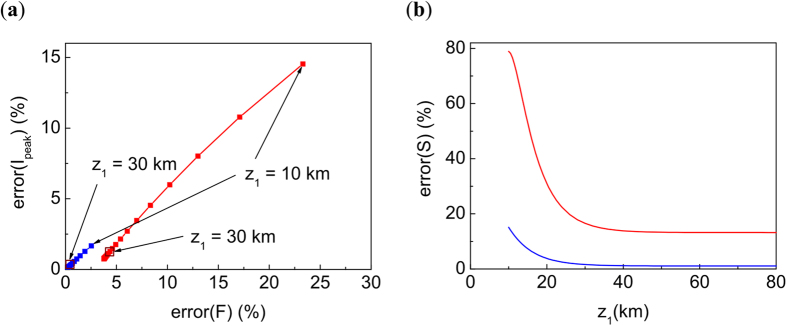
Determination of the optimal *z*_1_ = *C* = 5.93. (**a**) *error*(*F*) and *error*[*I*(0, *F*)] depending on *z*_1_, see Eqs (16 and 17). Plot points correspond to different values of *z*_1_ from 10 to 80 km. From point to point *z*_1_ varies with an increment step of 2 km. (**b**) *error*(*S*) depending on *z*_1,_ see Eq. (15). Blue line: *P*_*in*_/*P*_*cr*_ = 1000. Red line: *P*_*in*_/*P*_*cr*_ = 5000.

Figures 3, 4, 5, 6, 7 demonstrate that the results obtained by using the simple TW model are in good agreement with the direct NLSE simulations. In Fig. 3 we present the peak intensity distribution along z for the TW model of Eq. (4) and the corresponding NLSE solution. Here *h* = 0.000506, 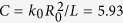, *L* = 1000 km, *R*_0_ = 1 m, *λ*_0_ = 1.06 μm and *P*_*in*_/*P*_*cr*_ = 1500 and 5000, with the corresponding factor nonlinear phase shift *b* = 3.02 or *b* = 10.05 respectively. Here and below (unless other values were mentioned specifically), the parameter *z*_1_ in the TW model was 30 km. In the numerical modelling we use (4) as the boundary condition for the linear propagation starting at *z*_0_ = 0.

Figure 3 shows an excellent agreement between the TW model and the full numerical simulations based on the NLS equation, both for evolution of the peak intensity with distance and for the radial beam intensity distribution. One can see that the TW model approximates well the exact solution of NLSE even in the situation with well-developed filamentation (see Fig.1b), with the field distribution being very far from the Gaussian beam. Note that in the TW model the solution depends on the dimensionless parameter 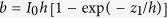, only, which simplifies the system optimization.

Let us discuss the calculations of the Strehl ratio. For the TW model (4), S can be calculated analytically, see Eq. (10). The results are not sensitive to *z*_1_ value. The comparison of the Strehl ratio computed with the NLSE solution (1) and the TW model (10) is plotted in Fig. 4a. We see that TW model is very close to NLSE solution, reproducing even non-monotonic S behavior. Let us stress again that the TW model is accurate for the calculation of intensity in spot center even at large *b*, when the beam is far from Gaussian.

The calculations of beam quality (*M*^2^) are presented in Fig. 4b. Within the TW model Eq. (4), *M*^2^ can be calculated analytically, as shown in Eq. (11). It is important to note that this result is valid for beams quite different from Gaussian. We see that the TW model provides an excellent description at modest b and slightly overestimates beam quality for large *b,* when the beam is already destroyed.

As we discussed above, the detrimental effects of self-focusing can be partially compensated by additional defocusing the beam by the quadratic (in radius) phase pre-distortion with the modified chirp. Mathematically, this means that instead of previous chirp parameter *C* we use modified *C*_*opt*_. Analytically, the optimal focusing parameter *C*_*opt*_ is a function of *b*. The optimal chirp gives the maximum intensity at the destination point *L* = 1000 km. The graph of the optimal *C*_*opt*_ as a function of *b* is presented in Fig. 5a. Here 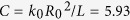, corresponding to a focal point *L*. The peak intensity at the initial focal point L corresponding to the initial chirp *C* and optimal chirp *C*_*opt*_ is presented in Fig. 5b. Figure 5c demonstrates that the TW model gives very accurate predictions for the shifted focal points *F*. Self-focusing changes the peak intensity, with the result depending on b as shown in Fig. 5d. We see that the initial defocusing can noticeably increase the Strehl ratio (see Fig. 5b), and that the TW model is good for predicting the optimal focusing parameters.

### Compensation of atmospheric aberrations by adaptive optics

The initial phase pre-distortion can be used to compensate the nonlinear phase changes. As a result, one can have an almost perfect Gaussian beam at the atmospheric exit and detrimental effects of self-focusing can be eliminated to a great extent. New initial condition with the corrected phase:


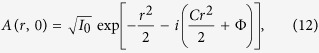



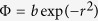
, 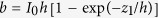, 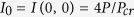.

We compare the solution of NLSE (1) with the initial condition (2) and chirp *C* = 5.93, which corresponds to the linear focusing at *L* = 1000 km (see, though, discussion in the^15^, around Equation 4); the solution of the same problem with optimal chirp 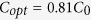; and the solution of NLSE (1) with a pre-imposed phase, phase (12); and the solution of the linear problem (5) with initial condition (2). The result is presented in Fig. 6. We see that the initial phase modification compensates nonlinear effects and the solution of NLSE is very close to the linear one. Note, that as expected, the solution of NLSE with pre-imposed phase correction (12) preserves the Gaussian shape in the transversal directions.

The above calculations used a simplified exponential model of the atmospheric density profile. The application of a more realistic profile does not affect the spatial structure of the phase Φ and changes the value of *b* only. The parameter *b* is an analogue of *B* integral for a non-uniform window. There is no need for the complete compensation of the phase, it is sufficient to ensure that the resulting *b* value is less than one. It is straightforward to demonstrate that the propagation is not really sensitive to *δb*, the difference between acquired and pre-imposed phase. Even for *b* = 10, variation of *δb*/*b* up to 0.1 practically doesn’t change the intensity structure in a focal plane in comparison with the complete compensation *δb* = 0.

We have demonstrated that the nonlinear effect of self-focusing in the atmosphere for space debris cleaning can be described with good accuracy within the thin window model. Within this model, the nonlinearity produces phase front distortion, serving as a high aberration focusing lens. Optical phase distortion results in displacement of the focusing point and beam filamentation, degrading the system performance. The former effect can be compensated partially by defocusing the initial beam. The TW model yields semi-analytical expressions for calculation of peak intensity on debris (Strehl number) and beam quality *M*^2^.

Typically, the nonlinear evolution of the self-focusing can be approximated as a fixed shape beam propagation with slowly varying parameters. The equations for these parameters can be derived from the Talanov virial theorem or from the variational principle^13^,^16^. Attempts to use this approach for the specific problem considered above failed due to beam filamentation and destruction. On the other hand, the TW model describes beam focusing with strong aberrations, with field distributions in the focal plane far from Gaussian. The pattern of laser field is determined by a single dimensionless parameter *b*, similar to the *B* integral used in laser design to control the self-phase modulation. The dependence on only one parameter greatly simplifies the optimization of the beam pre-focusing arrangements.

The description of linear propagation after exit from the atmosphere can be simplified using the fact that the field in the focal plane is proportional to the Fourier transform of the field exiting atmosphere. As a result, we obtained simple expressions for the peak intensity (Strehl ratio) and beam quality *M*^2^ which can be calculated in term of exiting field.

Because of the high accuracy of the TW model, one can compensate for self-focusing in the atmosphere by pre-imposed phase distribution which will cancel the nonlinear phase acquired during the propagation in atmosphere. Our modeling demonstrated that the detrimental effects off self-focusing can be almost completely eliminated by pre-imposed phase calculated within TW model.

## Conclusion

Removal of the space junk is a global issue that becomes more and more important. Methods of active de-orbiting defunct satellites are costly and technically demanding. Therefore, new approaches to efficient and effective clean-up of space debris are highly desirable. The nonlinear effect of high-power laser propagation in the atmosphere causes restrictions on ground-based laser systems for space debris cleaning. In general, the propagation of high power laser beams have already been discussed in the past, see e.g. refs 2,6,17,18. For the propagation of powerful CW beams the thermal blooming is a serious challenge^6^. Therefore, the pulsed (nanosecond scale) ground systems were put into the focus of research. In the regime of pulse propagation, there is no time for thermally induced modification of the refractive index and the blooming is not important. The thermal blooming can build up as a result of multiple pulses and limit the system repetition rate only. However, typically, the engineering restrictions on repetition rate are more severe. Also, the thermal blooming is proportional to laser absorption in air. Due to the many other reasons it is beneficial to build system at big elevation, with clean, low absorption environment.

The specific of the considered problem is that the beam propagates mainly in space, and the atmosphere only produces the phase aberration and all beam destruction is a pure linear effect. We have demonstrated that the thin window model provides high accuracy for light propagation and accurately predicts the phase distortion at the atmospheric edge and, thus, it can be very efficient in multi-parametric optimization of ground systems. Initial phase distortion can compensate the deviations produced by self-focusing, and can almost completely remove the detrimental effects of self-focusing. A similar approach can be used to compensate for the detrimental effects of self-focusing in femtosecond material processing. The development of powerful short pulse lasers was greatly advance by the National Ignition Facility construction and now the laser systems required for the ground base space debris cleaning are within the reach of the modern technology^3^. Another nonlinear effects (scattering by the atmospheric turbulence, Raman scattering) are detrimental for propagation and affects the optimal choice of system parameters^4^. Note, that recently the space based systems (see e.g. ref. 19) were proposed. They have no problem with atmospheric propagation. However, there are other specific challenges such as e.g. thermal management problems. The comprehensive studies are required to decide on the optimal roadmap to most promising future systems. We would like to stress that there are many challenges in this field related to technological implementations as well as atmospheric propagation that have been discussed in more detail e.g. in^19^,^20^. Our work should be considered in this general context and as a contribution to possible future solutions of this global problem.

## Methods

The propagation of the laser beam is modeled by the nonlinear Schrödinger equation (1) with an initially Gaussian laser beam (2). The equation is solved using the difference scheme with weights^21^,^22^:


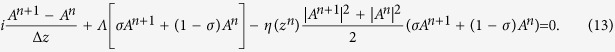


Laplacian 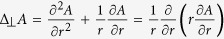 is approximated by a three-point stencil:


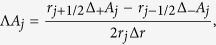


where 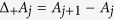 and 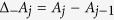 are forward and backward differences. The parameter *σ* is chosen as 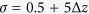 to achieve the stability of the scheme and the second order of accuracy 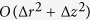.

Equation (13) is an implicit nonlinear scheme and is solved iteratively, similar to the two-step procedure used in the predictor–corrector schemes, for each fixed layer *n* on an evolutionary variable *z*. Specifically, we define the sequence 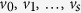 (s is the iteration number) that will converge to the solution *A*^*n*+1^ on the (*n* + 1)-th 1layer.

Since the thickness of the atmosphere is much smaller than the focusing length, the nonlinear effects are important only in a narrow layer, the remaining propagation is linear. Therefore, it is reasonable to use the additional iterations to linearize the scheme only at the initial steps of propagation. Numerical experiments show that the scheme can be used without the linearization procedure. Exclusion of the linearization affects the solution negligibly, but simplify the numerical method and reduce the computational time.

Equation (13) is solved in a dimensionless domain 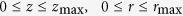, where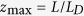, *L* = 1000 km, *L*_*D*_ = 11855 km, 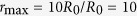. A uniform mesh is used with steps 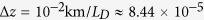, 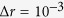. The numerical method described above is slightly different from the one presented in^21^,^22^, because here we have the Laplacian in polar coordinates that gives a singularity at the beam center *r* = 0. To define the boundary condition at this point we consider NLSE (1) in the Cartesian coordinates *x*,*y*:


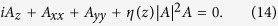


Equation (14) is approximated in the center of symmetry *x* = *y* = 0 with a mesh step 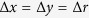. Second derivatives near zero:


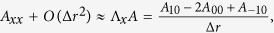
 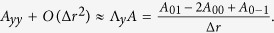


Since 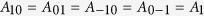 we have: 
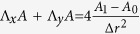
.

Thus, we approximate Eq. (14) using half-sums


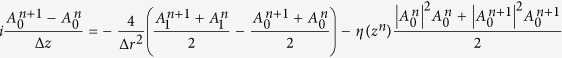


to get the order of accuracy 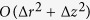. It is possibile to increase the order of accuracy of the scheme (13) to 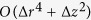 as described in^21^,^22^.

### Appendix

In this section we will discuss the accuracy of modeling using the TW model. We then check the accuracy of the model by the comparison of the field in the center of the beam in the initial focal plane


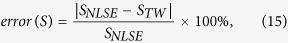


the displacement of the focal spot


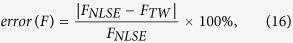


and the intensity maximum


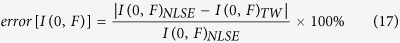


calculated with the exact NLSE and the TW model.

In Fig. 7 we present the error calculations for different beam powers. On Fig. 7a,b we present the results for *P*/*P*_*cr*_ = 1000 and 5000 as a function of *z*_1_. We see that as was discussed in the text the error is minimal for *z*_1_ ~ 30 km, justifying the choice of *z*_1_ used in the main text. We see that before the filamentation starts, for the modest values of b the accuracy is good. For high intensity, *P*/*P*_*cr*_ > 5000, the accuracy decreases but is still reasonable even for a completely filamented beam.

## Additional Information

**How to cite this article**: Vaseva, I. A. *et al.* Light self-focusing in the atmosphere: thin window model. *Sci. Rep.* **6**, 30697; doi: 10.1038/srep30697 (2016).
